# Deep-learning-based prognostic modeling for incident heart failure in patients with diabetes using electronic health records: A retrospective cohort study

**DOI:** 10.1371/journal.pone.0281878

**Published:** 2023-02-21

**Authors:** Ilaria Gandin, Sebastiano Saccani, Andrea Coser, Arjuna Scagnetto, Chiara Cappelletto, Riccardo Candido, Giulia Barbati, Andrea Di Lenarda

**Affiliations:** 1 Department of Medical Sciences, Biostatistics Unit, University of Trieste, Trieste, Italy; 2 Aindo, Trieste, Italy; 3 Cardiovascular Center, University Hospital and Health Services of Trieste, Trieste, Italy; 4 Diabetes Center, University Hospital and Health Services of Trieste, Trieste, Italy; King’s College London, UNITED KINGDOM

## Abstract

Patients with type 2 diabetes mellitus (T2DM) have more than twice the risk of developing heart failure (HF) compared to patients without diabetes. The present study is aimed to build an artificial intelligence (AI) prognostic model that takes in account a large and heterogeneous set of clinical factors and investigates the risk of developing HF in diabetic patients. We carried out an electronic health records- (EHR-) based retrospective cohort study that included patients with cardiological clinical evaluation and no previous diagnosis of HF. Information consists of features extracted from clinical and administrative data obtained as part of routine medical care. The primary endpoint was diagnosis of HF (during out-of-hospital clinical examination or hospitalization). We developed two prognostic models using (1) elastic net regularization for Cox proportional hazard model (COX) and (2) a deep neural network survival method (PHNN), in which a neural network was used to represent a non-linear hazard function and explainability strategies are applied to estimate the influence of predictors on the risk function. Over a median follow-up of 65 months, 17.3% of the 10,614 patients developed HF. The PHNN model outperformed COX both in terms of discrimination (c-index 0.768 *vs* 0.734) and calibration (2-year integrated calibration index 0.008 *vs* 0.018). The AI approach led to the identification of 20 predictors of different domains (age, body mass index, echocardiographic and electrocardiographic features, laboratory measurements, comorbidities, therapies) whose relationship with the predicted risk correspond to known trends in the clinical practice. Our results suggest that prognostic models for HF in diabetic patients may improve using EHRs in combination with AI techniques for survival analysis, which provide high flexibility and better performance with respect to standard approaches.

## Introduction

In the last decades Type 2 diabetes mellitus (T2DM) has become a global epidemic which is expected to affect over 592 million people worldwide by 2035 [1, 2]. Diabetes is associated with a decrease in life expectancy, in large part attributable to cardiovascular diseases [3]. In particular, patients with T2DM have more than twice the risk of developing heart failure (HF) compared to patients without diabetes mellitus [4, 5]. While 10% to 15% of the general population have diabetes, 44% of patients hospitalized for HF have diabetes mellitus [6]. The increased incidence of HF in diabetic patients persists even after adjusting for well-known risk factors for HF in general populations such as age, hypertension, hypercholesterolemia, and coronary artery disease. Therefore, in order to target high-risk individuals and reduce the risk of HF development with pharmacological agents (like sodium-glucose contransporter 2 inhibitor [7, 8]), the identification of diabetes-specific characteristics involved in HF development remains an important clinical question.

Several studies have developed clinical prognostic models for HF in diabetics patients, however they were not able to provide a comprehensive risk stratification and thus no score has yet been included in guideline care. In a recent work [9], Razaghizad et al. carried out a systematic evaluation on 15 models developed for hospitalization for HF in type 2 diabetes and showed that RECODe risk equation (together with TRS-HF_DM, another well performing model, although with a higher potential risk for bias) can be considered the most promising score for clinical use. Developed using data from the Action to Control Cardiovascular Risk in Diabetes study (ACCORD), the RECODe risk equations include age, sex, ethnicity, smoke, systolic blood pressure, history of cardiovascular disease, blood pressure-lowering drugs, statins, anticoagulants, HbA1c, total cholesterol, HDL, serum creatinine and urine albumin:creatinine ratio as predictors [10]. The model showed moderate-to-good discrimination and calibration in internal (c-index = 0.75, calibration slope = 1.01, intercept = -0.0004) and external validation (c-index = 0.76, calibration slope = 1.13, intercept = -0.011).

We hypothesized that an innovative approach based on the use of features available in electronic health records (EHRs) and artificial intelligence methods may improve performance in prognostic models in clinical settings. EHRs are the whole set of digital data originated at single-patient level in health care institutions as part of the clinical routine. Even though EHRs represent a valuable source of information (massive volumes, longitudinal nature, up-to-date, multiple domains) [11, 12], such data have been rarely include in risk scores because of low-quality issues, such as high heterogeneity and noise. Advances in artificial intelligence, in particular in neural networks architectures for deep learning, are offering computational techniques able to leverage the richness of EHRs for personalized healthcare [13–15]. Although deep learning approaches have been recently extended from prediction tasks to survival analysis, in which modeling right censored data is required [16–18], their application on EHRs for prognostic models has been limited.

In this study we investigate two research questions: (1) whether EHRs can improve risk stratification for HF in patients with type 2 diabetes; (2) the advantages of deep learning survival models over more standard approaches to account for non-linear effects and interactions between variables.

## Materials and methods

### Data

The present study is a cohort observational, retrospective study on patients enrolled in the Cardiovascular Observatory of Trieste (Italy) [19] affected by diabetes mellitus that had a cardiological evaluation from November 1, 2009 until December 31, 2018. All information on patients was extracted from clinical records and administrative data obtained as part of routine medical care. Data include medical information collected by cardiologists during routine clinical practice, diagnostic codes, laboratory tests, procedures, and cardiovascular drugs prescriptions sorted out using electronic indexes, comorbidities. Diagnosis of diabetes was based on multiple criteria: recorded diagnosis of diabetes, evidence of exemption for healthcare expenses, evidence of glycated hemoglobin levels>6.5%, purchase of at least two antidiabetic medications within one year. The first cardiological examination was considered the index visit, from which the absence of HF was ascertained (patients already diagnosed with HF when entering the Cardiovascular Observatory were excluded from the study).

### Endpoints

The primary endpoint was the onset of HF identified as the first between the following events: diagnosis of HF during hospitalization (ICD-9 codes: 39891, 40201, 40211, 40291, 40401, 40403, 40411, 40413, 40491, 40493, 4280–4284, 4289) and diagnosis of HF based at out-of-hospital clinical examination. Diagnosis of HF was performed according to ESC criteria: typical symptoms (breathlessness, ankle swelling and fatigue) and/or signs (elevated jugular venous pressure, pulmonary crackles and peripheral oedema) in presence of a structural and/or functional cardiac abnormality. Follow-up period for HF onset started at the index visit and ended on the administrative censoring date December 31, 2019. Death as a competing risk was not taken into account after an analysis of the Kaplan-Meier curve that showed a negligible bias within the first 60 months (S1 Fig). Baseline characteristics were compared between HF and HF-free individuals using chi-square test for categorical variables and t-test for continuous variables (or Mann-Whitney test, when appropriate).

### Derivation of the models

The cohort was randomly divided in training, validation and test set (70%, 15% and 15% respectively) maintaining approximately the same ratio of patients that experienced HF and censored patients. The test set was fixed beforehand and has been held out from the training of the different models tested and has been solely used to evaluate the models. We developed two models: first, a linear proportional hazards regression model (COX); second, a non-linear proportional hazards deep neural network model (PHNN).

Using elastic net regularization, a machine learning approach, we developed a Cox proportional hazard model that kept, among all possible covariates, only those identified as relevant predictors. According to the Cox hypothesis, the hazard function is assumed to be the product of two components *h*(*t*|***X***) = *h*_0_(*t*)*r*(***X***), where *h*_0_(*t*) is the baseline hazard function and *r*(***X***) is the risk function and approximated by the exponential of a linear function *r*(***X***) = *exp*(***β***⋅***X***). Elastic net regularization algorithm implements a Cox model via penalized maximum likelihood and it is able to select among candidate predictors the best model in the context of collinearity [20]. In our study L2 regularization parameter was set to 1 and 10-fold cross validation was performed to select the L1 regularization parameter λ following the “one-standard-error rule” [21] (given the context of application of the model and the need for a parsimonious model, we selected the largest value λ for which the CV error is within 1 standard error of the minimizing rule). Missing values were imputed using MICE, a multiple imputation technique based on chained equations [22].

In line with the work of Katzman et al. [16], we developed a second model that lifts the linear hazard hypothesis and model the risk function as *r*(***X***) = *exp*(*f*(***X***)), with *f*(***X***) a very generic function of the covariates. In particular, *f*(***X***) = *f*_*ϑ*_(***X***) has been approximated with a fully connected deep neural network (four layers with hidden size 128, 64, 32, 15 hidden units, rectifier non linearity, activation dropout 0.5 in all layers) parametrized by the weights *ϑ*. The standard package of auto-differentiation PyTorch was then used to minimize the partial likelihood over the train set to fit the network weights *ϑ*. Training was performed under early stopping using the validation set. To understand the dependence between variables and predicted risk, we reported the partial dependence plots (PDPs) [23] representing the marginal effect of covariates on predicted log-hazard. For a covariate of interest *X*_*k*_, and for any value *x*_*k*_ assumed by *X*_*k*_, we estimate the partial dependence function as the average $log\;\hat{h_{k}}(x_{k})=\frac{1}{n}\sum_{i=1}^{n}\hat{h}\left(x_{k},{\left\{{x_{j}}^{i}\right\}}_{j\neq k}\right)$ where *n* is the number of observations and *x*_*j*_^*i*^ is the value of covariate *X*_*j*_ for the *i*-th individual. Before training the model, a forward selection procedure was applied on the large number of variables included in the dataset. In the first step, a model was estimated using only one covariate at a time and the relative c-index was computed. The covariate with the highest c-index turned out to be “age”. In the second step, another round was performed using”age” and other variables one at a time. The feature corresponding to the model with highest c-index was then retained as the second feature. The process was repeated adding one feature at a time until adding new features did not bring a substantial improvement in the c-index. Notice that, to perform this feature selection, a validation set has been extracted from the training set, with the same size of the test set. This set was used to evaluate the many models considered, and not to train them. For the development of PHDL, missing values were imputed with the sample mean and a flag column was added to retain the information on values originally missing. Numerical columns were normalized to a normal distribution with a quantile transformer. In order to retain as much as possible information and to let the model learning from any hidden trend within the data, no additional feature engineering was applied. Validity of the proportional hazards assumption was checked using graphical diagnostics based on the scaled Schoenfeld residuals for all predictors selected by the models.

### Validation of the models

The discrimination of the models was evaluated by c-index (Harrell’s estimator) which is a generalization of the area under the receiver operating characteristic curve for time-to-event data and can be interpreted as the ability of a model to rank patients from high to low risk. Mean and standard error for the index was obtained through 10-fold cross-validation. In addition, time-dependent ROC curve was generated at 2- and 5-year of follow-up and the relative area under the curves (AUCs) were compared between models using inferential techniques [24]. We carried out graphical assessment of calibration by dividing subjects into 10 groups using deciles of the predicted probabilities and comparing predicted/observed risk across strata. Moreover, for each model we reported the Integrated Calibration Index (ICI) [25], which is the weighted average of the absolute difference between observed and predicted risks, in which the absolute differences are weighted by the empirical density function of the predicted risks.

We also compared predictions from our model with those from the RECODe study. In the calculation of RECODe equation, information on urine albumin:creatinine ratio was absent (as stated by the authors, individuals without a known covariate can have the relative term omitted from the equations). Moreover, instead of using the RECODe baseline HF-free survival reported in the article (0.96) to recalibrate the model we calculated an updated baseline HF-free survival by calculating the 2- and 5-year HF-free survival in the test set (0.927 and 0.856 respectively).

Analyses were done using R (version 4.2.1; R Foundation for Statistical Computing, Vienna) and Python 3.8.10 and PyTorch 1.10.2. The study involved the use of clinical records and administrative data produced as part of routine medical care, in compliance with the local regulatory and privacy policies. Data were collected in an anonymous form. A written informed consent was obtained under the institutional review board policies of hospitals administration. The current study was approved by Comitato Etico Unico Regionale FVG (Protocol ID: 114_2020T). All information was linked and anonymized before the analysis.

## Results

The cohort included 10,614 patients: 4,447 (42%) were females and mean age was 72 (SD = 11). Baseline characteristics according to heart failure diagnosis are presented in Table 1. During a median of 65 months, 1840 patients (17.3%) developed HF. Probability of HF-free survival at 2- and 5-year was 92.7% (95% CI [94.8,95.6]) and 85.6% (95% CI [84.9,86.3]) respectively.

**Table 1:** Characteristics of the cohort separately for individuals free from HF and individuals that have developed HF. GFR was estimated using the EPI-CKD formula. History of cardiovascular disease includes stroke and myocardial infarction. SD = standard deviation; IQR = interquantile range; RASi = Renin–angiotensin system inhibitors; MRA = Aldosterone receptor antagonists.

Clinical characteristics	HF free (n = 8774)	HF (n = 1840)	p-value
Age, years	72 (64, 79)	77 (71, 82)	<0.001
Median (IQR)			
Male gender	3,700 (42%)	747 (41%)	<0.001
N (%)			
BMI, kg/m2	28.7 (23.6, 33.8)	28.7 (23.4, 34.0)	0.8
Mean (±SD)			
Systolic blood pressure, mmHg	140 (120, 160)	141 (120, 162)	0.005
Mean (±SD)			
Diastolic blood pressure, mmHg	80 (70, 90)	80 (70, 90)	0.2
Mean (±SD)			
Heart rate, b.p.m.	72 (64, 82)	72 (63, 82)	0.7
Median (IQR)			
GFR, mL/min	78 (62, 90)	67 (52, 83)	<0.001
Sodium, mEq/L	139.2 (136.1, 142.3)	139.4 (136.4, 142.4)	0.013
Mean (±SD)			
Hemoglobin, g/DL	13.49 (11.64, 15.34)	13.12(11.23 15.01)	<0.001
Mean (±SD)			
Glycated hemoglobin, %	6.70 (6.24, 7.50)	6.78 (6.20, 7.50)	0.8
Cholesterol, mg/dL	184 (136, 232)	180 (133, 227)	0.004
Mean (±SD)			
HDL, mg/dL	48 (33, 63)	47 (31, 63)	0.7
Mean (±SD)			
Triglycerides, mg/dL	127 (85, 186)	123 (81, 179)	0.018
Median (IQR)			
Creatinine, mg/dL	0.89 (0.74, 1.10)	0.98 (0.79, 1.23)	<0.001
Median (IQR)			
Smoking status	1,098 (13%)	183 (9.9%)	0.002
N (%)			
History of cardiovascular disease	1,572 (18%)	416 (23%)	<0.001
N (%)			
Atrial fibrillation	1,155 (13%)	443 (24%)	<0.001
N (%)			
Hypertension	6,582 (75%)	1,646 (89%)	<0.001
N (%)			
Obesity	2,152 (25%)	545 (30%)	<0.001
N (%)			
Peripheral artery disease	860 (9.8%)	306 (17%)	0.2
N (%)			
Chronic kidney disease	2,042 (23%)	703 (38%)	<0.001
N (%)			
Chronic obstructive pulmonary disease	432 (4.9%)	167 (9.1%)	<0.001
N (%)			
Anaemia	638 (7.3%)	205 (11%)	<0.001
N (%)			
History of cerebrovascular accident	929 (11%)	269 (15%)	<0.001
N (%)			
‍Metformin	3,047 (35%)	711 (39%)	0.001
N (%)			
Antihypertensives	5,529 (63%)	1,401 (76%)	<0.001
N (%)			
RASi	4,536 (52%)	1,135 (62%)	<0.001
N (%)			
Digitalis	152 (1.7%)	110 (6.0%)	<0.001
N (%)			
Beta-blocker	2,936 (33%)	807 (44%)	<0.001
N (%)			
MRA	293 (3.3%)	175 (9.5%)	<0.001
N (%)			
Statines	3,757 (43%)	840 (46%)	0.026
N (%)			
Anticoagulants	632 (7.2%)	269 (15%)	<0.001
N (%)			
Diuretics (loop)	567 (6.5%)	433 (24%)	<0.001
N (%)			
Diuretics (other)	2,338 (27%)	815 (44%)	<0.001
N (%)			
Duration of diabetes, months	69 (16, 129)	91 (23, 131)	<0.001
N (%)			
Organ damage	1,904 (22%)	687 (37%)	<0.001
N (%)			

**Fig 1:**
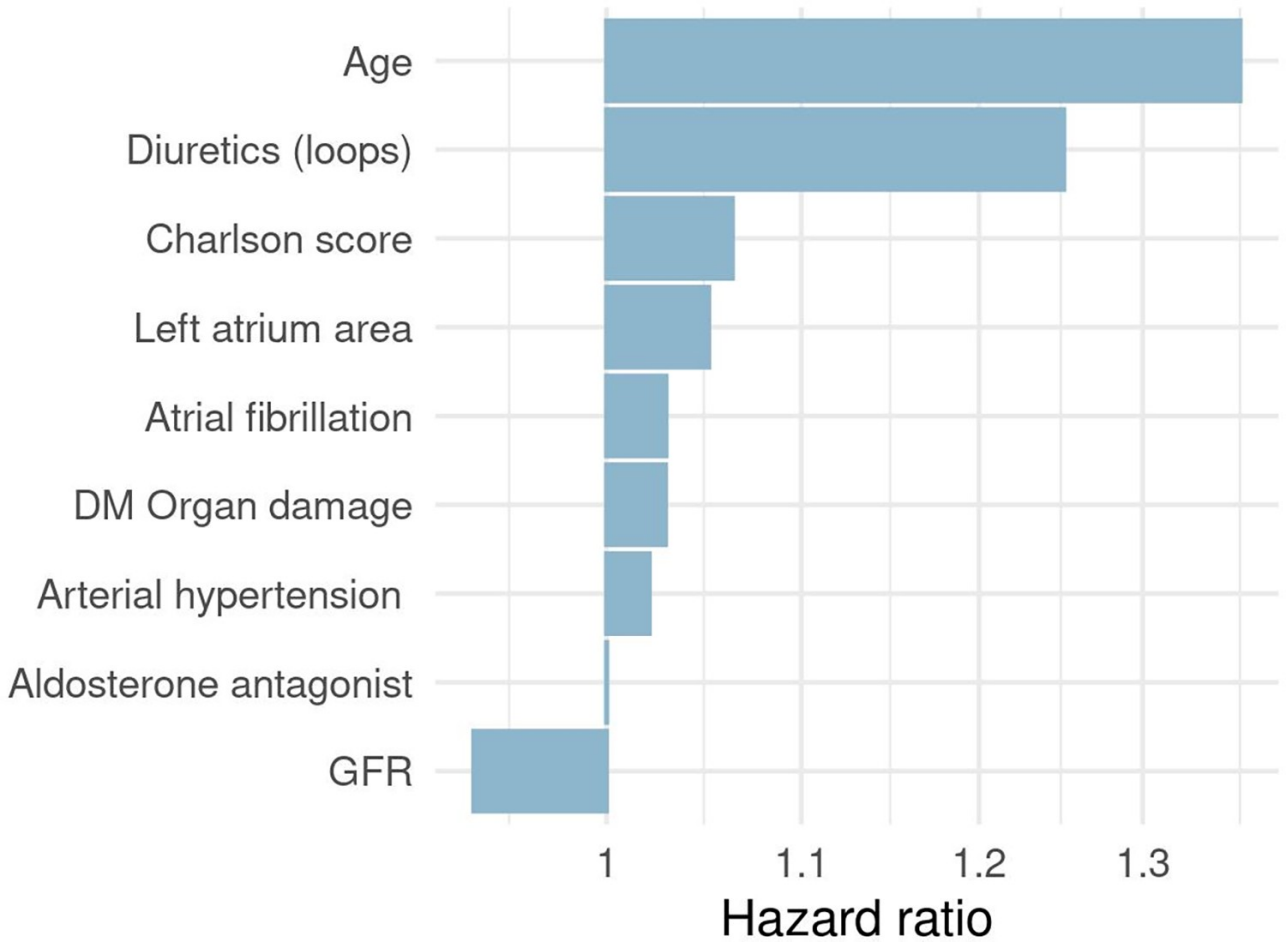
Predictors included in the penalized Cox model ordered by magnitude of effect. GFR is the only variable associated with hazard ratio<1, meaning that higher values of GFR are associated with lower risk of HF.

Based on elastic net regularization, most relevant variables for predicting HF were age, diuretics, Charlson score, left atrium area, atrial fibrillation, organ damage, hypertension, adolsterone antagonist, glomerular filtration rate (protective factor) (Fig 1).

**Fig 2:**
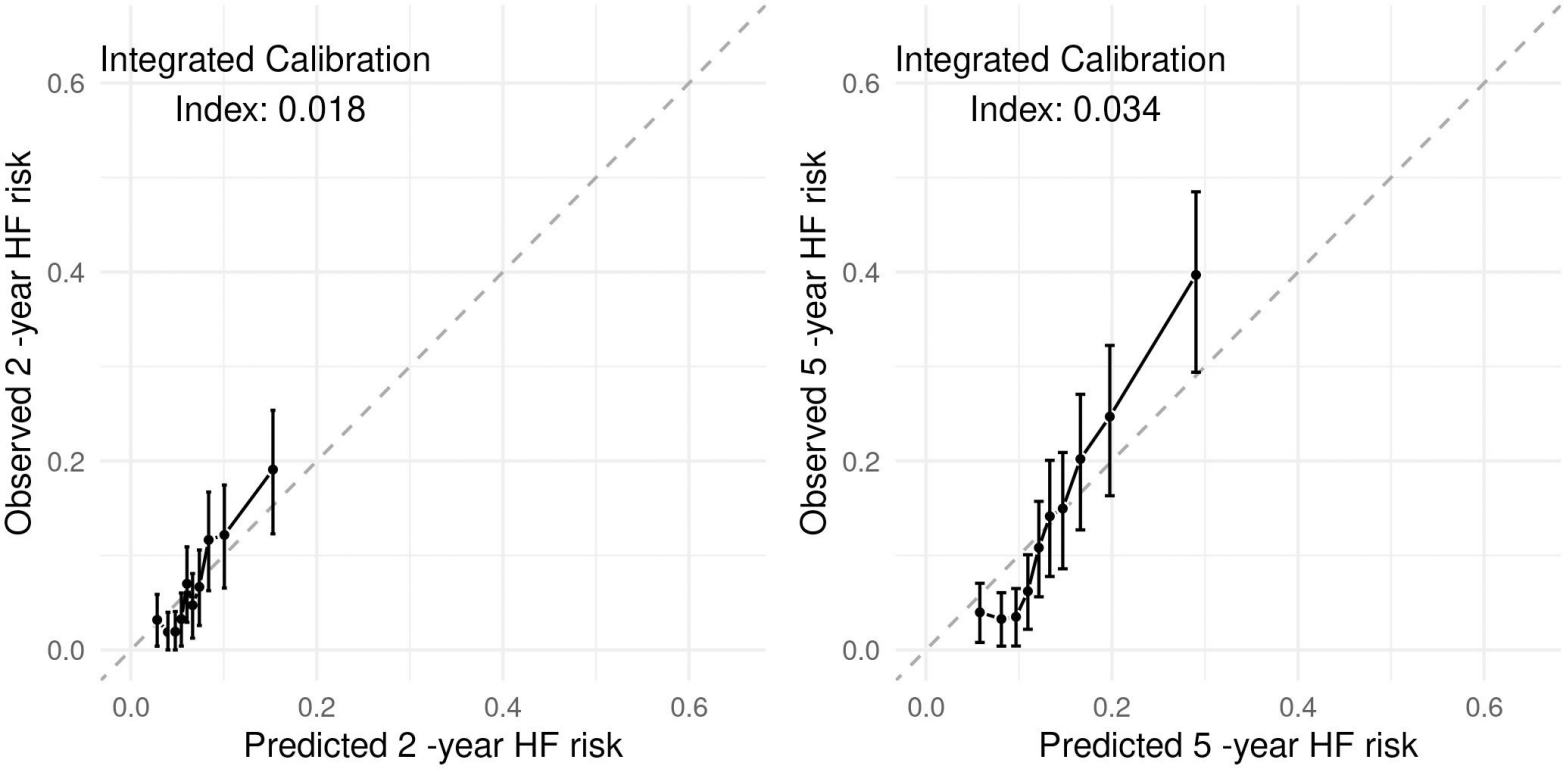
Calibration for COX model. Deviations from the diagonal line denote lack of calibration.

Using the penalized Cox model we obtained a c-index of 0.734 (Table 2). Considering time points 2 years and 5 years, the area under the time-dependent ROC was 0.716 (95% CI [0.664,0.767]) and 0.770 (95% CI [0.732,0.807]) respectively. As depicted in Fig 2, calibration of prediction was acceptable at 2 years (ICI = 0.018) but considerably decreased for predictions at 5 years (ICI = 0.034).

**Table 2:** Performance of the three models. SE = standard error; AUC = Area under the ROC curve; ICI = integrated calibration index; CI = confidence interval.

	C-index	2-year AUC	2-year ICI	5-year AUC	5-year ICI
	±SE	[95% CI]		[95% CI]	
**COX**	0.734	0.716	0.018	0.770	0.030
	±0.004	[0.664,0.767]		[0.732,0.807]	
**PHNN**	0.768	0.771	0.008	0.780	0.015
	±0.007	[0.723,0.818]		[0.743,0.817]	
**RECODe**	0.670	0.651	0.715	0.668	0.533
		[0.601,0.701]		[0.621,0.709]	

**Fig 3:**
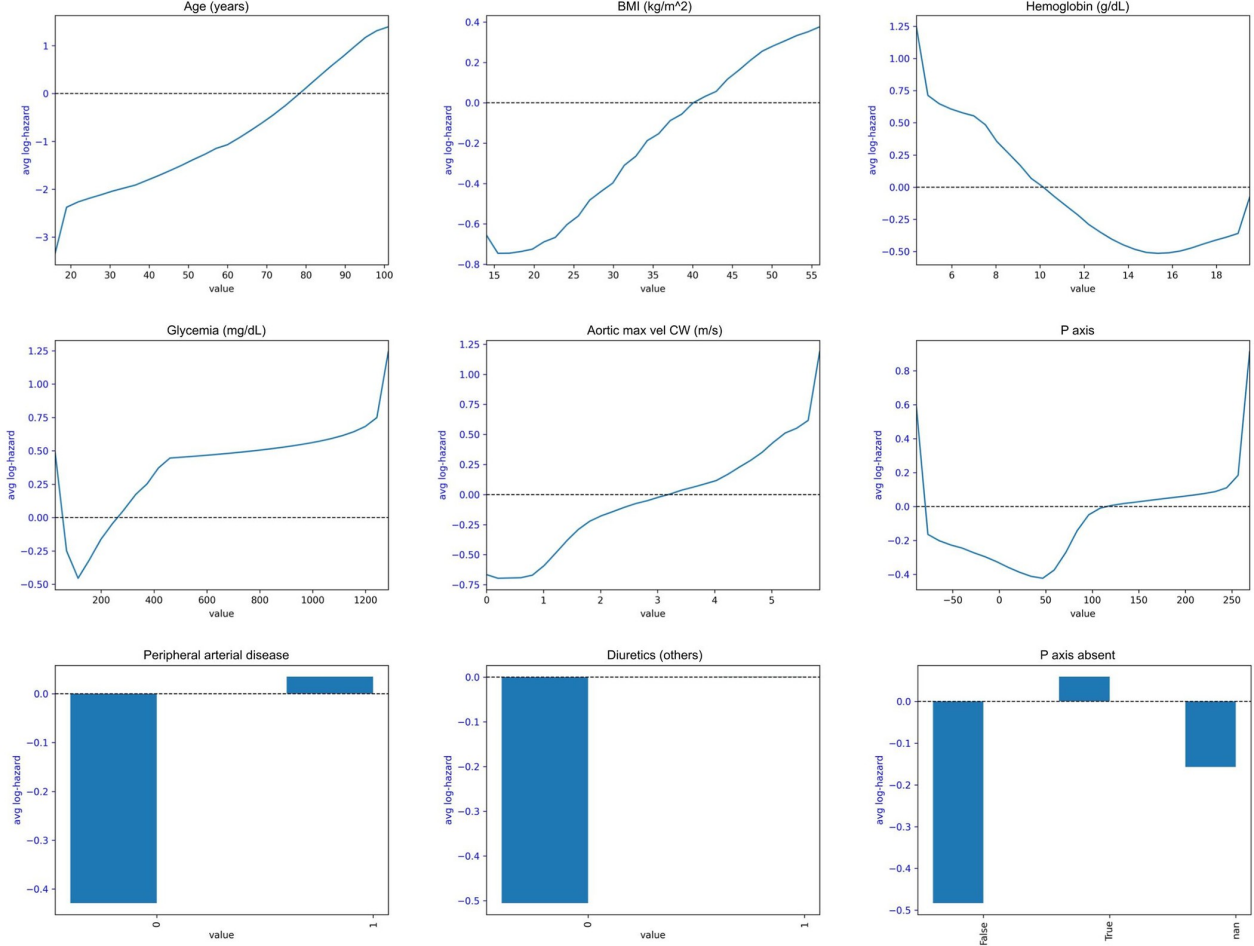
Partial dependence plots for PHNN model. The blue line (or bar for categorical values) represents the value of the log hazard for various values of the covariate (x axis). Higher values correspond to higher hazard.

As for the DL approach, the feature selection process identified 20 relevant variables (S2 Fig, S1 Table): age, BMI, four echocardiographic parameters (left ventricular wall motion score index, continuous wave aortic velocity, tissue doppler E wave velocity, tricuspid regurgitation), three ECG parameters (P axis absent, P axis, T axis), five comorbidities (renal disease, hypertension, lung disease, pericardium disease, peripheral artery disease), three laboratory measurements (hemoglobin, glycemia, triglyceride levels) and three categories of medication (diuretics, anticoagulants, RASi). In Fig 3 and S3 Fig it is possible to observe for each predictor the partial dependence plot, representing the relationship between the variable and the log-hazard.

**Fig 4:**
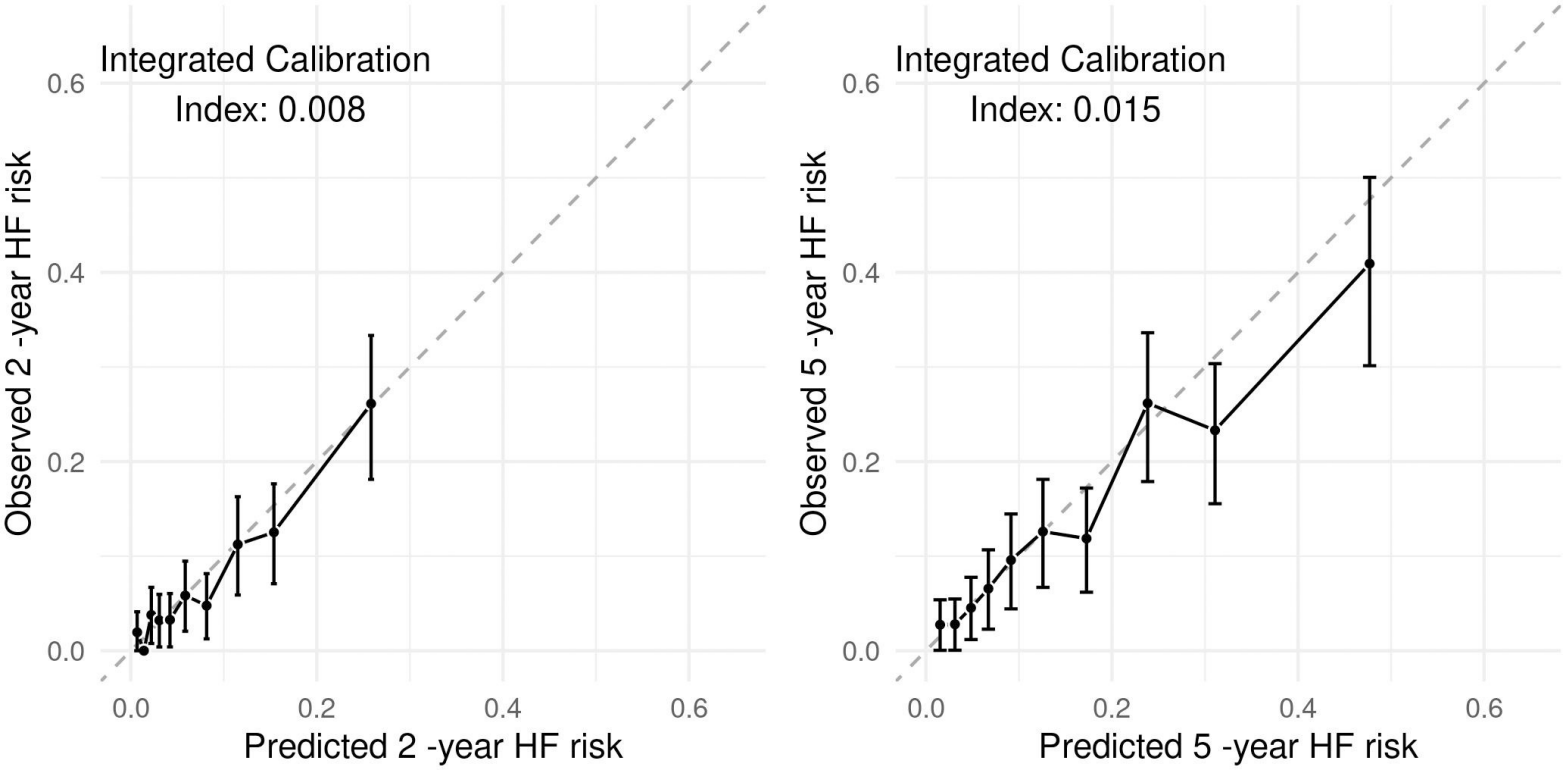
Calibration for the PHNN model.

Using the DL model we obtained a better discrimination, with a c-index of 0.768. In the time-dependent ROC analysis we observed better accuracy as well: AUC was 0.771 (95% CI [0.723,0.818]) at 2-year of follow-up and 0.780 (95% CI [0.743,0.817]) at 5-year of follow up, although differences were not statistically significant (2-year p-value = 0.059, 5-year p-value = 0.772). Moreover, the ICI for 2- and 5-year risk (0.008 and 0.015, respectively) indicated good calibration (Fig 4). Additional information on models performance is reported in S2 Table.

Compared with our models, RECODe risk score had worse discrimination (c-index of 0.670, see Table 2). In particular, predicted survival was less accurate compared to the penalized Cox model both at 2-year follow-up (AUC = 0.651, 95% CI [0.601,0.701], p-value = 0.042) and 5-year follow-up (AUC = 0.668, 95% CI [0.621,0.709], p-value<0.001). Significant decrease in discrimination was observed also with respect to the DL model (2-year p-value<0.001, 5-year p-value<0.001).

## Discussion

We developed two prognostic models for HF in diabetic patients using EHRs (one assuming linear hazard and the other assuming a non-linear hazard) and showed the benefit of implementing deep learning algorithms in terms of performance.

Among previous studies that developed prognostic models for HF risk in patients with diabetes, RECODe risk equations are considered the most promising for clinical use. However, such model performed poorly in our cohort. A possible explanation can be found in the characteristics of our cohort: we applied the model in a clinical setting that included patients with a wide range of comorbidities, unlike individuals part of the research cohorts in which the model was derived.

Both our models were superior to RECODe risk equations. COX model obtained with elastic net regularization identified eight clinical predictors already associated with HF risk. As reported in [9], diabetes duration, diuretics, atrial fibrillation, arterial hypertension, are risk factors commonly included in prediction models for HF, along with GFR as protective factor. Charlson comorbity index [26] was found to be a risk factor for heart failure readmission in another large-scale study [27]. One of the most relevant predictors was loop diuretics (HR = 1.25), which are commonly used in the treatment of HF, thus possibly a sign of presence of masked HF cases not detectable from EHRs [28]. Model’s discrimination ability was acceptable but calibration showed poor results (in particular for 5-year risk). This could be due to the low flexibility in the model’s specification.

On the other side, the implementation of a deep neural network in the PHNN model made possible to reach moderate performance in terms of discrimination and well-calibrated predictions. As for variable selection, eight of the PHNN covariates were either predictors of RECODe equations (age, systolic blood pressure, blood pressure-lowering drugs, anticoagulants) or common risk predictors reported in [9] (BMI, hemoglobin, chronic kidney disease, peripheral artery disease). It is interesting to notice that one of the COX predictors was diagnosis of atrial fibrillation: such variable was not included in the PHNN model, however in the list of predictors we observe “absence of P axis” and “P axis” that is an ECG feature closely related with atrial fibrillation. Moreover, one of the predictors was continuous wave aortic velocity which high values could indicate aortic stenosis, a condition that often coexists with atrial fibrillation and predispose to HF [29]. Others relevant echocardiographic parameters were: 1) tissue doppler E wave velocity, for which we estimated an inverse proportional relationship with HF risk; 2) tricuspid valve regurgitation, showing an increase in HF risk in case of moderate-to-severe regurgitation; and 3) wall motion score index, proportionally related with HF risk. Regarding laboratory measurements, hemoglobin and glycemia showed a well-known U-shape effect; whereas triglycerides exhibit an unexpected trend, for which HF risk decreases for higher values. Concerning comorbidities, the model included chronic kidney disease, hypertension, pulmonary disease and peripheral artery disease as risk factors, while pericardium disease as protective factor. Moreover, three categories of therapies influenced the HF risk: use of diuretics (other than loop diuretics) and anticoagulants as risk factors, RASi use as protective factor. The T axis, an ECG feature also included in the model, has no straightforward interpretation.

Although our neural network model showed a limited gain in the identification of diabetic patients that are going to develop HF, an improvement in the performance that can not be considered clinically significant, we demonstrated the feasibility of using EHRs and AI to approach the prognostic problem and obtained consistent results with respect to the clinical knowledge in the field. Moreover, our deep learning model showed adequate calibration, an important aspect of a predictive model that not always couples with discriminative ability. In particular, recent advances in deep learning methods have demonstrated incredible gains in prediction accuracy, but producing well-calibrated probabilities remains a challenge for AI tools [13, 30, 31]. In fact, this is one of the major obstacles for the use of AI tools in clinical practice for personalized medicine, since using uncalibrated predictions to determine a patient’s individual risk could led to incorrect medical decisions [32]. Our results could be relevant for future developments of prognostic risks, with a view to integrated cardiovascular prediction tools. The utility of AI applied to massive raw datasets, like ECG and echocardiograms, is being demonstrated as a powerful tool for phenotyping of cardiac conditions that can be employed at the point of care [33, 34]. In the case of ECGs, recent studies have introduced tools combining deep representations of data obtained from convolutional neural networks (in substitution to manual feature engineering) with EHRs variables [35, 36]. In the same way, for survival analysis, employing deep learning models represents the most promising and feasible way to operate in ultrahigh dimensional settings (eg. signals and images), a task that standard modeling strategies (including regularization methods) simply can not undertake. A key concern with DL approaches is the lack of transparency, since the inner-workings of such models is intrinsically a “black-box”. However, we believe that the development and application of advanced explainability techniques can provide relevant information on models’ behavior and could contribute to build the trustworthiness required for their usage in the clinical practice, as we expressed in a recent work [15].

The present study has some limitations. First, the cohort in examination is formed by individuals that underwent a cardiological evaluation. Even if cardiological assessment is highly recommended for diabetic patients, in our cohort we can not exclude the presence of selection bias towards individuals with higher cardiovascular risk with respect to the general diabetic population. In addition to this, the PHNN model include ECG and echocardiographic parameters as predictors, that can be obtained during a cardiological evaluation and this limit the applicability of the model to patients that were visited by a cardiologist. Second, our model was not validated in independent external cohorts. Future studies should be directed to measure the performance in one or more independent cohorts. Third, using explainability techniques, we are able to study and describe the marginal effect of single predictors, however we have no information on the interaction effects that could have an important role in the determination of predicted risk. Forth, in the current setting of the study possible changes in predictors variables during follow-up are not taken in account. The proposed model should be intended only as a prognostic tool at the basal evaluation.

## Conclusion

In this study we create a prognostic tool for the management of diabetic patients at risk of developing incident HF using an AI approach that leverages the potential of EHRs. This approach may also be extended to other sources of data, like signals (ECGs) and images (echocardiography, magnetic resonance imaging).

## Supporting information

S1 Fig In blue, cumulative incidence function (CIF). In red, 1—Kaplan-Meier curve.
(PDF)

S2 Fig Features selection for the PHNN model. Blue bars correspond to C-index obtained using the single variable. The red line corresponds to the cumulative performance on the validation set adding one variable at the time.
(PDF)

S3 Fig Partial dependence plots for PHNN model. (PDF)

S1 Table Missing rate of predictors involved in the models. (PDF)

S2 Table Sentivity, specificity, positive predicted value, negative predicted value for COX and PHNN model for 2- and 5-year predictions. Values refer to the cut-off level that obtained the higher value in the Youden’s index (Youden WJ. Index for rating diagnostic tests. Cancer 1950;3(1):32–5).
(PDF)
