# Supplementary figures and images for: Deep-learning-based prognostic modeling for incident heart failure in patients with diabetes using electronic health records: A retrospective cohort study
Source: PLoS One. 2023 Feb 21;18(2):e0281878. doi: 10.1371/journal.pone.0281878 (PMC9943005; doi:10.1371/journal.pone.0281878)

Supporting information

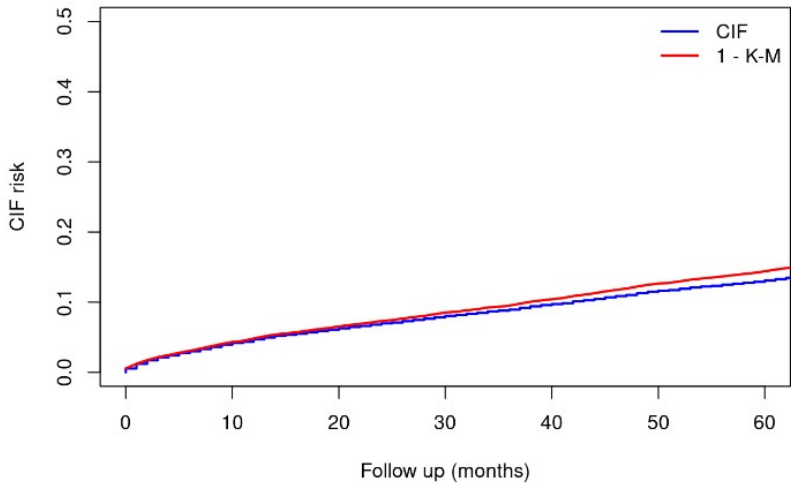

Figure S1.

Supplement: S1 Fig — In red, 1—Kaplan-Meier curve. (PDF) [file pone.0281878.s001.pdf]

Supporting information

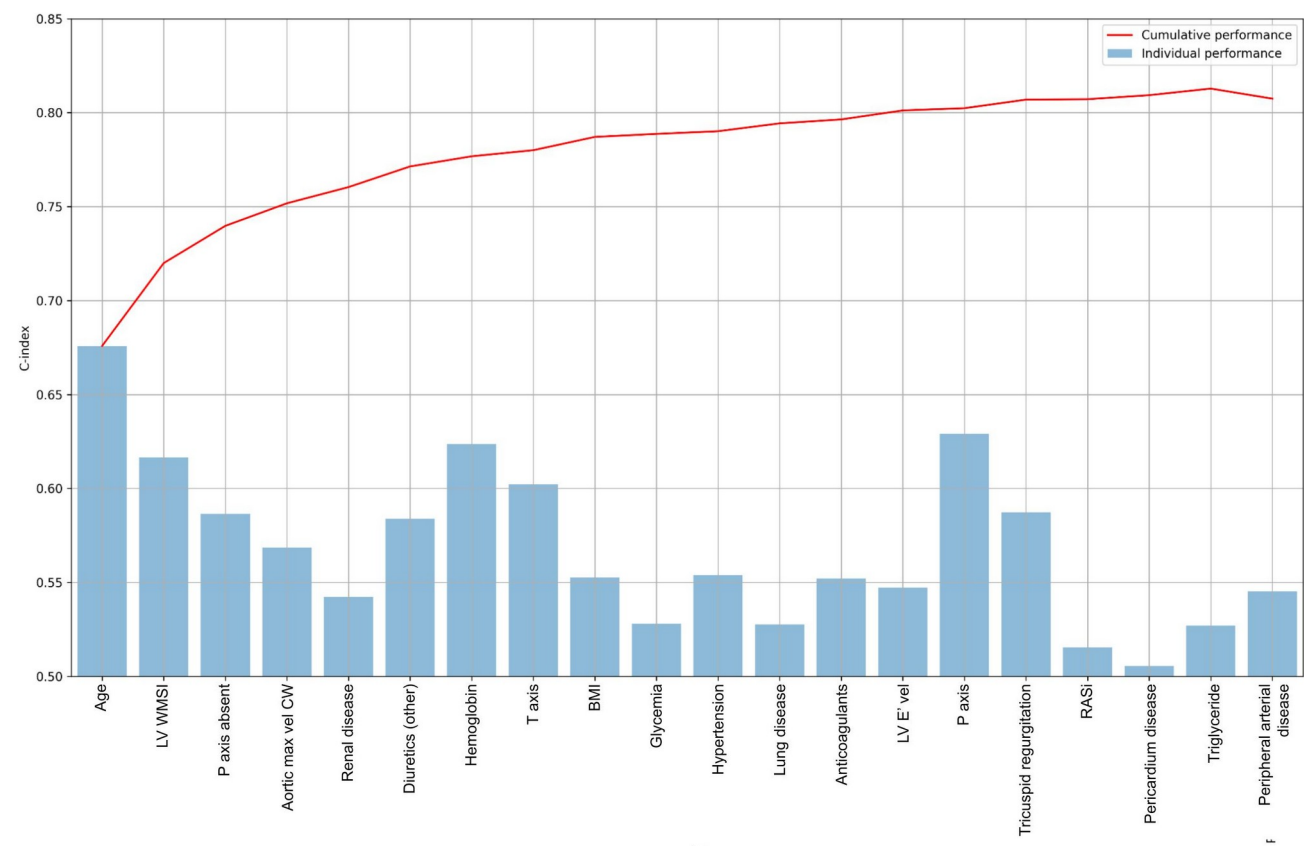

Figure S2.

Supplement: S2 Fig — Blue bars correspond to C-index obtained using the single variable. The red line corresponds to the cumulative performance on the validation set adding one variable at the time. (PDF) [file pone.0281878.s002.pdf]

Supporting information

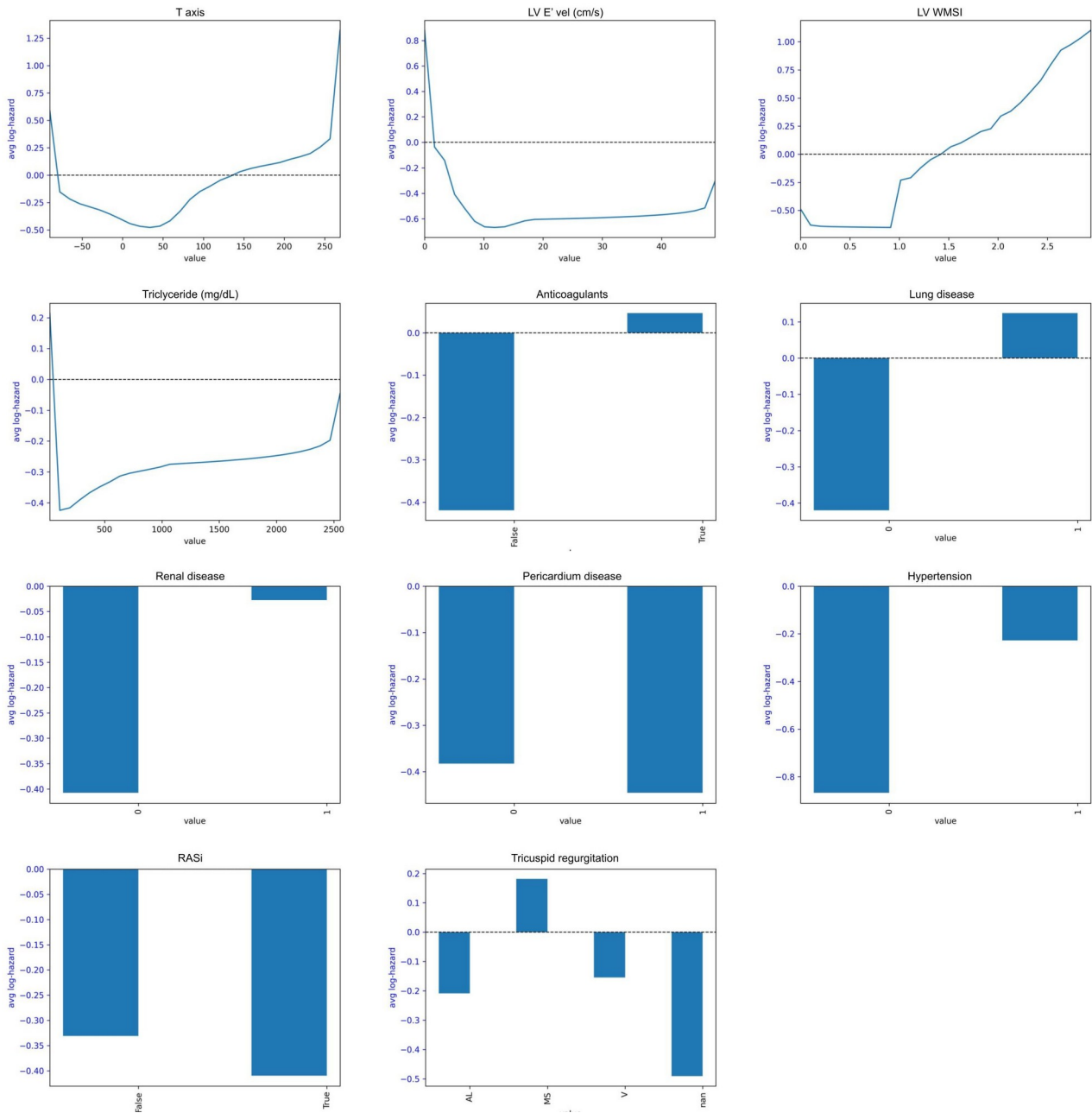

Figure S3.

Supplement: S3 Fig — (PDF) [file pone.0281878.s003.pdf]
