# Supplementary material for: Deep-learning-based prognostic modeling for incident heart failure in patients with diabetes using electronic health records: A retrospective cohort study
Source: PLoS One. 2023 Feb 21;18(2):e0281878. doi: 10.1371/journal.pone.0281878 (PMC9943005; doi:10.1371/journal.pone.0281878)
Supplement: S1 Table — (PDF) [file pone.0281878.s004.pdf]

Supporting information

**Variable**	**Missing rate (%)**
Age	0
Aldosterone antagonist	0
Anticoagulants	0
Arterial hypertension	0
Atrial fibrillation	0
BMI	20
Charlson score	0
Continuous wave aortic velocity	62
Diuretics (loops)	0
DM Organ damage	0
Diuretics	0
GRF	11
Glycemia	15
Hemoglobin	13

Hypertension	0
Left ventricular wall motion score index	51
Lung disease	0
P axis	13
P axis absent	0
pericardium disease	0
peripheral artery disease	0
RASi	0
renal disease	0
T axis	13
Tissue doppler E wave velocity	78
Tricuspid regurgitation	0
triglyceride levels	43

Table S1.
