# Supplementary material for: Deep-learning-based prognostic modeling for incident heart failure in patients with diabetes using electronic health records: A retrospective cohort study
Source: PLoS One. 2023 Feb 21;18(2):e0281878. doi: 10.1371/journal.pone.0281878 (PMC9943005; doi:10.1371/journal.pone.0281878)
Supplement: S2 Table — Values refer to the cut-off level that obtained the higher value in the Youden’s index (Youden WJ. Index for rating diagnostic tests. Cancer 1950;3(1):32–5). (PDF) [file pone.0281878.s005.pdf]

Supporting information

Model	Cutpoint	Sensitivity	Specificity	Positive predicted value	Negative predicted value
COX<br>24 months	0.156	0.634	0.706	0.137	0.963
COX<br>60 months	<0.001	0.776	0.627	0.240	0.949
PHNN<br>24 months	0.368	0.671	0.761	0.171	0.969
PHNN<br>60 months	0.173	0.671	0.760	0.298	0.938

Table S2.
